# Quantifying the Effects of Elastic Collisions and Non-Covalent Binding on Glutamate Receptor Trafficking in the Post-Synaptic Density

**DOI:** 10.1371/journal.pcbi.1000780

**Published:** 2010-05-13

**Authors:** Fidel Santamaria, Jossina Gonzalez, George J. Augustine, Sridhar Raghavachari

**Affiliations:** 1 Biology Department, The University of Texas at San Antonio, San Antonio, Texas, United States of America; 2 Neurosciences Institute, The University of Texas at San Antonio, San Antonio, Texas, United States of America; 3 Department of Neurobiology, Duke University Medical Center, Durham, North Carolina, United States of America; University of Houston, United States of America

## Abstract

One mechanism of information storage in neurons is believed to be determined by the strength of synaptic contacts. The strength of an excitatory synapse is partially due to the concentration of a particular type of ionotropic glutamate receptor (AMPAR) in the post-synaptic density (PSD). AMPAR concentration in the PSD has to be plastic, to allow the storage of new memories; but it also has to be stable to preserve important information. Although much is known about the molecular identity of synapses, the biophysical mechanisms by which AMPAR can enter, leave and remain in the synapse are unclear. We used Monte Carlo simulations to determine the influence of PSD structure and activity in maintaining homeostatic concentrations of AMPARs in the synapse. We found that, the high concentration and excluded volume caused by PSD molecules result in molecular crowding. Diffusion of AMPAR in the PSD under such conditions is anomalous. Anomalous diffusion of AMPAR results in retention of these receptors inside the PSD for periods ranging from minutes to several hours in the absence of strong binding of receptors to PSD molecules. Trapping of receptors in the PSD by crowding effects was very sensitive to the concentration of PSD molecules, showing a switch-like behavior for retention of receptors. Non-covalent binding of AMPAR to anchored PSD molecules allowed the synapse to become well-mixed, resulting in normal diffusion of AMPAR. Binding also allowed the exchange of receptors in and out of the PSD. We propose that molecular crowding is an important biophysical mechanism to maintain homeostatic synaptic concentrations of AMPARs in the PSD without the need of energetically expensive biochemical reactions. In this context, binding of AMPAR with PSD molecules could collaborate with crowding to maintain synaptic homeostasis but could also allow synaptic plasticity by increasing the exchange of these receptors with the surrounding extra-synaptic membrane.

## Author Summary

One of the most accepted theories of information storage in neurons is that it is partially localized in the strength of synaptic contacts. Evidence suggests that at the cellular level, in combination with other cellular mechanisms, this is implemented by increasing or decreasing the concentration of a particular type of membrane molecules. Two opposing mechanisms have to coexist in synapses to allow them to store information. On one hand, synapses have to be flexible, to allow the storage of new memories. On the other hand, synapses have to be stable to preserve previously learned information. Although much is known about the molecular identity of synapses, the biophysical mechanisms by which molecules can enter, leave and remain in the synapse are unclear. Our modeling work uses fundamental biophysical principles to quantify the effects of molecular collisions and biochemical reactions. Our results show that molecular collisions alone, between the diffusing proteins with anchored molecules in the synapse, can replicate known experimental results. Molecular collision in combination with biochemical binding can be fundamental biophysical principles used by synapses for the formation and preservation of memories.

## Introduction

Ligand-gated neurotransmitter receptors in the post-synaptic membrane respond to neurotransmitter release and thereby mediate rapid signaling at neuronal synapses. Efficient synaptic signaling demands that these receptors be concentrated at high densities in order to optimally respond to rapidly diffusing neurotransmitter molecules. For instance, at excitatory glutamatergic synapses of the central nervous system, alpha-amino-3-hydroxy-5-methyl-4-isoxazolepropionic acid receptors (AMPARs) are highly concentrated (1000 receptors/µm^2^) relative to the extra-synaptic membrane (2.7 receptors/µm^2^) [1], [2]. AMPARs are concentrated in a large membrane-associated protein complex called the post-synaptic density (PSD) [3]. The current model of AMPAR aggregation into the PSD comprises of several steps. First, AMPARs are trafficked to the synapse [4] where they are inserted in the extra-synaptic membrane via exocytosis [5]. While in the extra-synaptic membrane AMPARs undergo lateral diffusion and are randomly captured by the PSD through direct and indirect biochemical interactions with multiple partners [6], [7], [8], [9], [10], [11]. Within the PSD, AMPARs continue a more restricted diffusion process [10], [12], [13]. Upon unbinding, receptors can diffuse out of the PSD and are then recycled via endocytosis or targeted for degradation. Although AMPARs bind to scaffolding proteins in the PSD it seems that biochemical interactions alone cannot explain the retention of AMPARs required to achieve long lasting changes in synaptic strength. For example, genetic manipulation of PSD scaffolding protein levels does not abolish basal synaptic transmission and leaves the amplitude of spontaneous excitatory potentials unchanged [14], [15], [16].

About 42% of the PSD mass is composed of proteins that do not necessarily bind to AMPAR [3]. The presence of large numbers of high-molecular weight proteins significantly restricts protein diffusion within cells [17], [18], an effect known as macro-molecular crowding [19]. Molecular crowding can cause a breakdown of the laws of mass transport by causing anomalous diffusion of macro-molecules, i.e. the mean-squared displacement (MSD) of the molecule is no longer linear over time [20]. Anomalous diffusion due to molecular crowding is a physical process fundamentally different from considerations of excluded volume at steady-state, such as tortuosity [21], [22]. Molecular crowding affects not only the diffusion of molecules but also their reaction kinetics [17], [23]. Structural and imaging studies suggest that the PSD is likely a crowded environment, such that even lipids undergo confined diffusion [24], [25]. If molecular crowding affects the diffusion of AMPAR, this would require a reassessment of the biophysical mechanisms that control the homeostatic and dynamic concentration of these receptors in the PSD.

Here we used Monte Carlo simulations to study the effects of PSD molecular crowding on AMPAR concentration. We found that the expected levels of molecular crowding in the PSD result in anomalous diffusion of receptors and are capable of reproducing the characteristic non-linear dependence of the MSD of AMPARs as a function of time. Our simulations indicate that the extent of AMPAR trapping in the PSD is a switch-like function of the concentration of PSD molecules. We also studied receptor accumulation after synaptic stimulation by allowing PSD molecules to bind to AMPARs [11], [26], [27]. Binding allowed AMPARs trapped in a crowded PSD to increase their net mobility [24]. In summary, our results suggest that molecular crowding can maintain homeostatic synaptic concentrations of AMPARs without requiring energetically expensive biochemical modifications, while binding to PSD proteins allows synaptic plasticity by increasing the mobility of these receptors.

## Results

The objective of this work was to understand the physical mechanisms that maintain and regulate the concentration of synaptic AMPAR in the PSD. Because we were studying mechanisms in which classical theories of diffusion and reaction might not be applicable [22], [28], [29], [30], [31], it was necessary to study this process at the level of individual molecules [32], [33] instead of the more widely used mass-action formalism [7], [8], [34], [35].

### AMPAR density in the PSD maintained by molecular crowding

**Figure 1:**
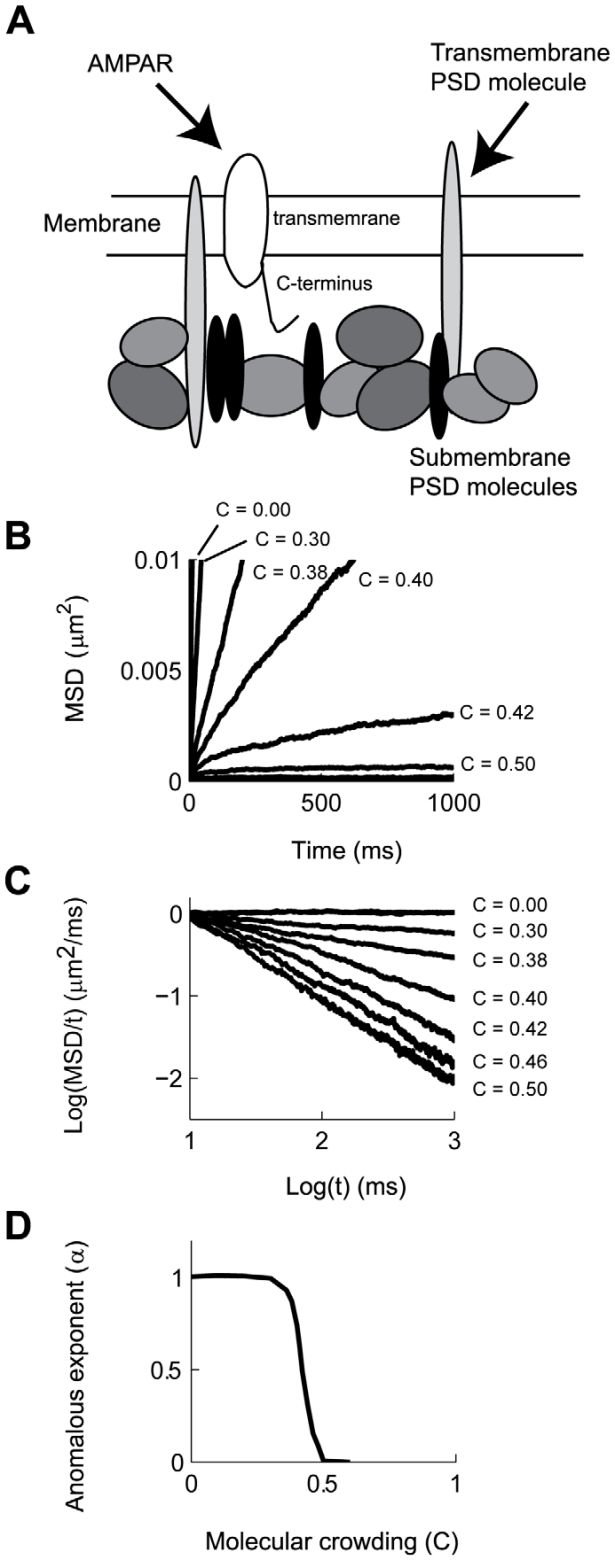
AMPAR diffusion is reduced by collisions with anchored PSD molecules. **A**: Schematic representation of AMPAR interactions in the PSD. **B**: MSD vs t plots of AMPAR diffusing in a membrane with increasing fraction occupied by anchored PSD molecules (C). **C**: Logarithmic transform of the data presented in **B**. The plots show that the classical law of diffusion (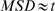) is replaced by anomalous diffusion (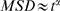), with 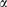 decreasing as a function of C. Each curve is calculated from 400 simulations. **D**: Calculated value of α from linear fits to the data presented in **C**.

The PSD of glutamatergic synapses is characterized by a high density network of scaffolding proteins located below the membrane, as well as trans-membrane proteins including receptors, ion channels and adhesion molecules. All of these molecules are linked together into a coherent structure by extensive protein-protein interactions [3]. Moreover, several PSD molecules, such as PSD-95, 4.1N, and AKAP, can undergo lipid modification that allows them to intercalate into the plasma membrane, bringing them close to the cytoplasmic domains of diffusing receptors [36], [37]. Thus, AMPAR diffusion can be sterically hindered by PSD molecules even in the absence of direct binding (Fig. 1A).

We simulated the diffusion of receptors within synaptic and extra-synaptic regions over a discrete lattice of 2×2 µm, with periodic boundary conditions to obviate finite size effects (see Materials and Methods). The large concentrations of macro-molecules such as the one found in the PSD, the cytoplasm or membrane of cells is referred to as molecular crowding [17]. In our models, we represented molecular crowding as populating a fraction (C) of grid points occupied by PSD molecules, such that at C = 0, no obstacles are found on the membrane and an AMPAR can freely diffuse (with diffusion coefficient D_free_). We first studied the effects of molecular crowding of PSD molecules on the movement of AMPARs in a membrane in the case where the AMPARs do not bind to PSD molecules. Classical theories of diffusion suggest that the effect of elastic collisions with static obstacles is to reduce the D_free_ of AMPAR to a lower constant value known as the apparent diffusion coefficient (D_app_) [17], [22]. This effect is referred to as tortuosity [38]. Instead, we found that this relationship grew increasingly non-linear as a function of C (Fig. 1B). Diffusion under such conditions is described by a different diffusion equation known as anomalous diffusion [18], [39], [40]:
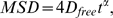
(6)where 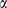 is the anomalous exponent. Clearly, the power law dependence is linear when plotting the logarithm of the MSD/time against the logarithm of time (Fig. 1C)
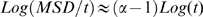
(7)The value of 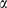 depends on the value of C (α = α(C)), such that at C = 0 diffusion is unobstructed and the dependency between MSD and time follows a linear relationship characteristic of normal diffusion (eq. 6 with α = 1). Thus, experimental measurements that quantify α can determine whether diffusion is normal (α = 1) and therefore can be analyzed with traditional mass action formalism, or whether diffusion is anomalous (α<1) showing strong deviations from traditional steady-state analysis [40].

Further analysis of the dependence of α as a function of C showed a switch-like behavior. The sigmoidal shape of the plot shown in Fig. 1D can be divided into three regions. The first region, C<0.3, shows a small effect of molecular crowding on AMPAR movement, resulting in normal diffusion (α = 1). Conversely, for C>0.5 the value of α is practically zero, indicating that AMPAR movement is severely reduced, but still possible. In this case, AMPARs are continuously colliding with PSD molecules being trapped in very small pockets within the PSD. Upon escaping one pocket, the diffusing receptors fall into a different area of high density of PSD molecules where they again continue to diffuse in a restricted manner [18]. For intermediate values, 0.3<C<0.5, there is a steep decrease in the value of α, with the corresponding deviation from normal diffusion. It is important to note that the fractional power-law dependency of the MSD vs. *t* is not expected from classical theories of diffusion or by assuming tortuosity. Tortuosity is characterized by a constant diffusion coefficient (α = 1) that is lower than what would be expected from unobstructed diffusion [22]. In anomalous diffusion there is no characteristic time constant for the system and the apparent diffusion coefficient decays with time [18], [40], [41], making α the appropriate physical variable to describe a diffusion process under such conditions.

**Figure 2 pcbi-1000780-g002:**
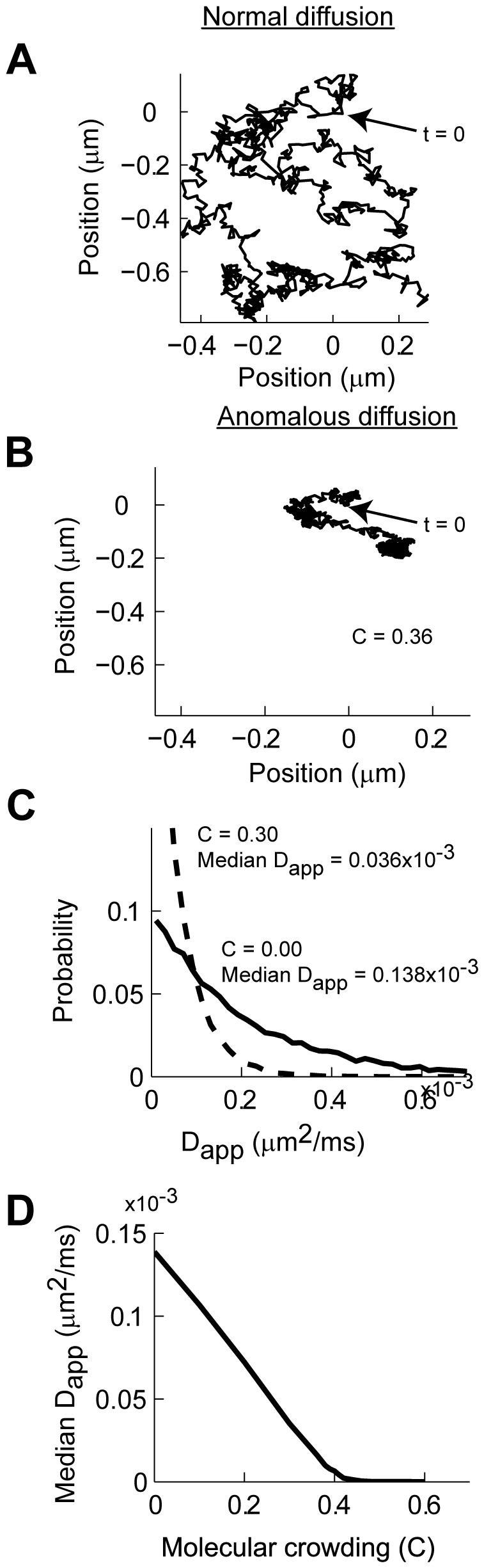
Molecular crowding affects the diffusion of AMPAR in the PSD. **A**: An example of a trajectory of a simulated AMPAR in a membrane free of obstacles (C = 0.00). **B**: An identical simulation as in A with the same initialization of the random number generator for an AMPAR diffusing on a membrane covered by fixed PSD molecules with a molecular crowding of C = 0.30. **C**: The calculated histogram of apparent diffusion coefficients (D_app_) with no obstacles (C = 0, dashed line) results in a median value of D_app_ = 0.138×10^−3^ µm^2^/ms. A similar curve calculated with C = 0.36 results in a median D_app_ = 0.038×10^−3^ µm^2^/ms (solid line). **D**: Median D_app_ as a function of molecular crowding calculated for all the simulations shown in Figure 1.

There are multiple physical consequences that arise from anomalous diffusion, from increases in temporal correlation of the position of the diffusing particles to changes in biochemical reactions rates [40]. The effect of molecular crowding results in a process where no equilibrium of concentration is achieved over the duration of our simulations [19]. Diffusing over a random field of PSD molecules results in randomly distributed areas where AMPARs move almost freely, while in other areas the steric interactions practically confine movement without the need for binding to other proteins [18]. The trajectories that diffusing particles follow under anomalous diffusion differ from the ones measured under normal conditions (Fig. 2A and B) [10]. While trajectories under normal diffusion visit the entire membrane (Fig. 2A), particles undergoing anomalous diffusion spend more time in some areas than in others (Fig. 2B). In order to relate our simulations to experimentally measured quantities, we calculated the instantaneous D_app_. In this case, D_app_ = Δx^2^/4Δt, where Δx is the displacement from the initial position after a set time Δt determined by the observer. Under conditions of free diffusion (C = 0) the histogram of values of D_app_ results in a median D_app_ equal to that reported experimentally (Fig. 2C solid line) [10]. In contrast, under diffusion under anomalous diffusion due to PSD molecular crowding the histogram of D_app_ is reduced sharply, resulting in a higher probability of finding AMPARs with extremely low D_app_ even in the case of moderate values of molecular crowding (C = 0.36, Fig. 2C dashed line) [10], [32]. The median value of D_app_ decays rapidly as a function of molecular crowding (Fig. 2D). Qualitatively, the distribution of D_app_ observed in our model resembles that reported experimentally using single particle tracking of AMPARs in neurons [10], [13]. Overall, the analyses shown in Fig. 1 and 2 suggest that diffusion inside synapses can be severely hampered by molecular crowding. The effects of molecular crowding can obscure the contribution of binding to the retention of AMPARs in the PSD and could also contribute to the retention of glutamate receptors inside the synapse.

**Figure 3 pcbi-1000780-g003:**
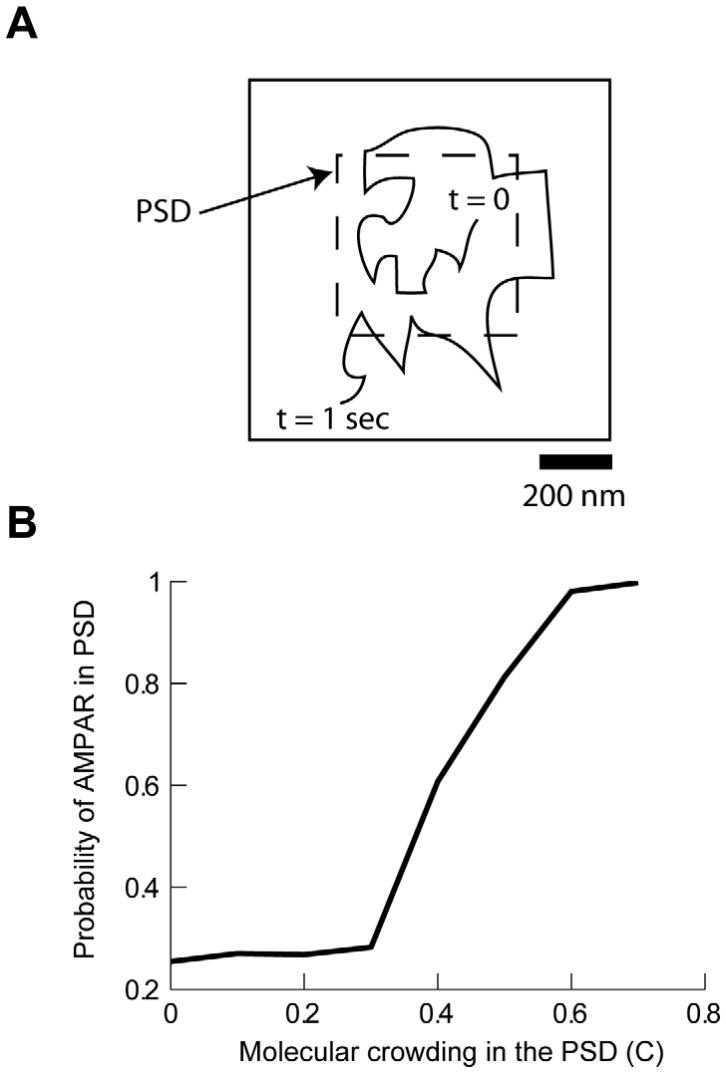
Molecular crowding in the PSD traps AMPAR in the synapse. **A**: Schematic diagram of the simulation paradigm. At t = 0 AMPAR are instantiated in the region inside the PSD and their position is recorded at t = 1 sec. The simulation consisted in releasing AMPAR inside the area of PSD and counting the relative probability of being trapped in that area as a function of the fraction of PSD molecules (molecular crowding). The PSD was a rectangle of 500 nm in size. **B**: Relative probability of finding AMPARs inside the PSD as a function of PSD molecular crowding (C). Each data point was calculated with 400 simulations.

Since the effects of increasing molecular crowding could be classified as binding to static PSD molecules, we examined the potential contribution of the PSD molecules to the retention of AMPARs inside the PSD. We simulated the diffusion of AMPARs on a square PSD 0.5 µm in width [24] placed in the center of a 1 by 1 µm membrane with toroidal boundary conditions to avoid finite-size effects. In each simulation an AMPAR was released at a random position inside the boundaries of the PSD. After 1 sec, we determined whether an AMPAR remained inside or escaped outside the area occupied by the PSD (Fig. 3A). Fig. 3B shows how the probability of finding a receptor within the area of the PSD varies according to the fraction of that area occupied by PSD molecules (C). At C = 0 the probability of a receptor residing within the PSD is, as expected, equal to the fraction of the membrane occupied by the PSD (0.25). For C>0.6, AMPARs initially positioned inside the PSD remain confined there. Within a concentration range of 0.3–0.5, the relative trapping of receptors showed a very steep dependence on small changes in PSD molecule concentration. In this regime, AMPAR can still move in and out of the PSD, but their net movement is significantly slowed by molecular crowding. As a consequence, the time spent inside the PSD is extended, a result similar to that produced by a model of receptor dynamics based on molecular interactions [42]. The difference between the two models is that our model does not require biochemical interactions between protein receptors and PSD scaffold molecules. We note that a random distribution of PSD molecules is essential in generating anomalous diffusion; a regular distribution of obstacles increases tortuosity but yields normal diffusion (see Figs. 1 and 2 in Text S1). Therefore, these results indicate that the steady-state concentration of AMPAR in the PSD can be maintained by physical confinement of AMPARs due to molecular crowding within the PSD.

**Figure 4:**
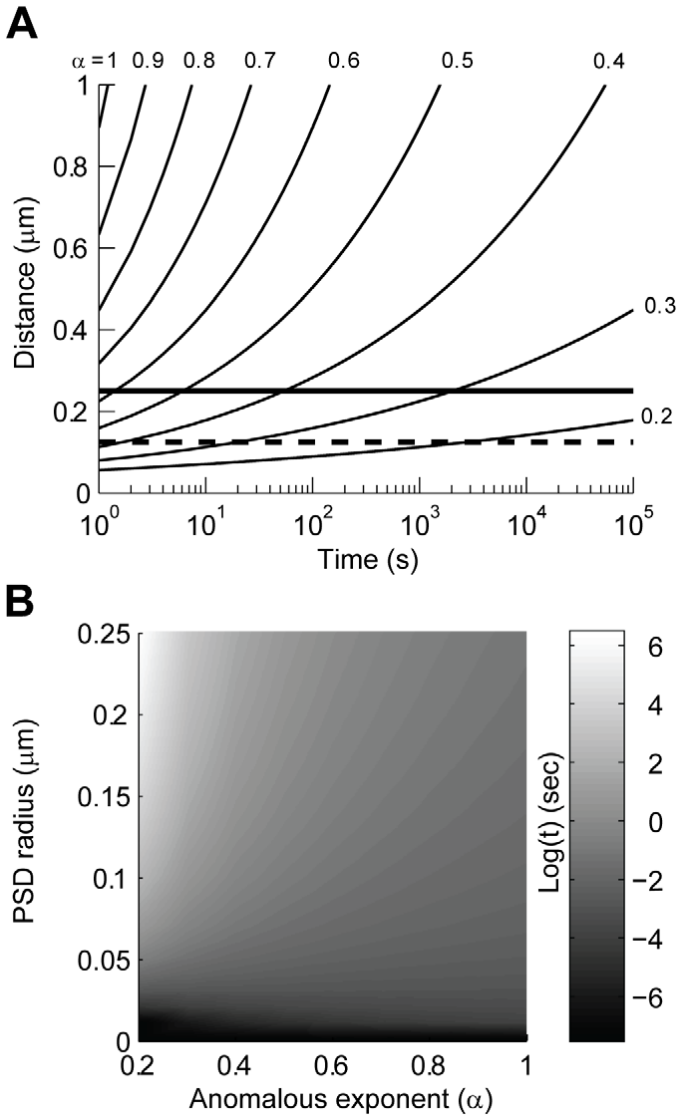
Anomalous diffusion could retain AMPARs inside a PSD for very long periods of time. **A**: Distance as a function of time for a molecule undergoing anomalous diffusion. The plots were calculated using the anomalous diffusion equation (distance = √(4 D_free_t^α^), D_free_ = 0.200×10^−3^ µm^2^/ms). We assumed an AMPAR released in the center of a typical PSD, the dashed line corresponds to the average diameter of a PSD (125 nm), while the thick line is for a large PSD (250 nm). **B**: Gray-scale plot of the Log(t) calculated using the same equation as in A. The plot shows the time a particle takes to reach the edge of a PSD after being released in the center. We calculated this value for a wide range of PSD sizes and anomalous exponent (α).

The time that an AMPAR is trapped inside the PSD depends on the amount of molecular crowding and position with respect to the PSD boundary. Equation 6 can be used to predict the time an AMPAR released in the center of a PSD would take to reach the boundary and escape. Using D_free_ as the diffusion coefficient and a range of 0.3<α<0.4, which corresponds to a minute difference in molecular crowding from C = 0.44 to 0.42, an AMPAR would reach the boundary of an average PSD of 125 nm in 4–76 seconds (denoted by the dashed line in Fig. 4A); which is in very good agreement with experimental observations [10], [13], [43]. However, retention time is very sensitive to PSD radius; for example, in a large PSD (250 nm radius) AMPARs will remained trapped for 100–10,000 seconds (2.4 minutes to 2.15 hours; solid line in Fig. 4A). Further analysis shows the sensitivity of residence time to PSD diameter and the value of α (Fig. 4B). Thus, if molecular crowding has an effect in the movement of AMPAR, small changes due to re-arrangements of the PSD, insertion of transmembrane proteins, or conformational changes that allow steric interactions, could dramatically influence the time an AMPAR remains trapped inside a PSD without the need for strong biochemical interactions with scaffold proteins.

The model originally developed by Kusumi et al. [44] is widely used to analyze the diffusion of AMPARs [10], [13], [24], [43], [45]. As opposed to our model, Kusumi's model assumes that a diffusing transmembrane protein is tethered to a loose cytoskeleton or is physically confined into an area bounded by reflecting walls. This model determines the confinement length (L) of the area explored by a molecule as it diffuses (MSD = (L^2^/3)(1−exp(−10D_free_t/L^2^)))). If the assumption is that the molecules are confined to diffuse within compartments, then the calculated diffusion coefficient is equal to D_free_. We fitted Kusumi's model to the MSD plots obtained in Fig. 1. This analysis indicates that for a molecular crowding of C = 0.40 the confinement length is L = 250 nm, which corresponds to the classified ‘slow’ moving AMPARs in PSDs [24]. This same group report the presence of ‘fast’ moving AMPAR with L = 600 nm, which corresponds to a value of C = 0.38 (see Fig. 3 in Text S1). Interestingly, the mobility of AMPAR increases with the application of glutamate, resulting in an increase of the diameter of area explored (from 225±3 nm during control to 239±15 nm during glutamate stimulation). Consistent with our hypothesis that steric interactions in the PSD hinder diffusion this same study shows that lipids also undergo confined diffusion inside the PSD [24]. It is important to note that ‘slow’ (or immobile) and ‘fast’ moving particles naturally emerge as part of the same process of anomalous diffusion [46]. We fitted eq. 6 to several reported curves of MSD against time for extra-synaptic and synaptic AMPAR diffusion [10], [24], [43], [45]. Remarkably, for diffusion inside synapses most of the fits resulted in crowding values close to C = 0.44, while for extra-synaptic diffusion C∼0.40 (Fig. 4 in Text S1). Overall, our analyses in Figs. 1–4, and Figs. 3 and 4 in Text S1 suggest that molecular crowding in the PSD could be of the order of C = 0.40–0.46 and that a small change in crowding could significantly regulate the mobility of AMPARs. The value of C for synaptic diffusion obtained from fitting eq. 6 to experimental data is in good agreement with calculations of excluded volume based on the spatial dimensions and density of PSD proteins (see Discussion).

### Receptor-scaffold interactions in the PSD as a mechanism for loading synaptic AMPAR

Synaptic activity regulates the number of AMPAR and their mobility in and out of the PSD [10]. This regulation of AMPAR density inside the PSD could be direct, with synaptic activity leading to unbinding or binding of AMPAR complexes to PSD molecules; or could be indirectly caused by spatial rearrangement of the PSD structure, with the resulting change in molecular crowding allowing AMPARs to move in and out of this structure. To understand the relative contributions of binding and crowding to AMPAR regulation in the PSD, we initially measured the effects of increasing the fraction of PSD molecules (P) that could bind to AMPAR.

**Figure 5:**
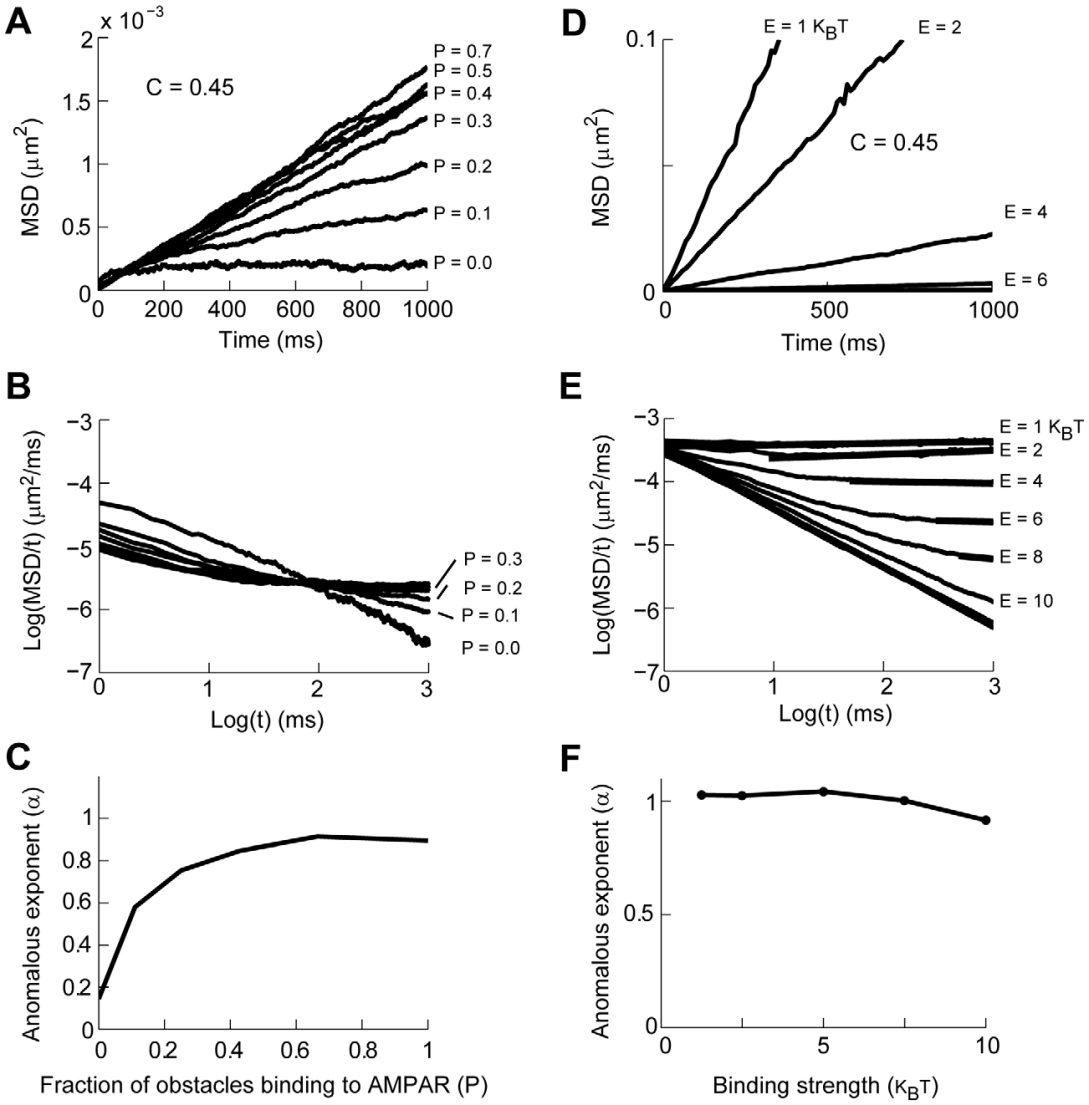
The effects of binding and molecular crowding on AMPAR diffusion in the PSD. **A–C**: Binding to PSD molecules allows AMPAR diffuse over a molecularly crowded membrane. **A**: MSD vs t plot of AMPAR diffusion over a membrane covered by PSD molecules at a density of C = 0.45. When all molecules are obstacles diffusion is practically stopped. As the fraction of PSD molecules that bind to AMPAR increases (P) the mobility of the receptor increases. Binding energy range from 4–8 k_B_T. **B**: Logarithmic transform of the data in **A** shows that for P = 0 AMPAR are trapped in the PSD. As P increases AMPAR diffusion shows different values of anomalous diffusion. **C**: Calculation of the anomalous exponent as a fraction of the obstacle molecules binding to AMPAR. **D–F**: AMPAR diffusion where all PSD molecules can bind to AMPAR. **D**: MSD vs t plot of AMPAR diffusion over a membrane covered by PSD molecules at a density of C = 0.45. All PSD molecules can bind to AMPAR with an identical binding energy (E). **E**: Logarithmic transform of the data in D shows that diffusion is anomalous for a brief period of time to then become normal (thick lines). **F**: The anomalous exponent along the linear part of the plots in **E** as a function of binding energy (k_B_T).

Independent of the precise binding and unbinding probabilities of AMPAR to other proteins or the potential multi-step nature of receptor-scaffold interactions, the range of values for hydrogen bonds for protein-protein interaction motifs such as the PDZ domains is 2–13 k_B_T [47], [48]. Figure 5 shows the results of simulations in which the membrane was covered by PSD molecules with a density of C = 0.45 and the binding energies for the interaction of PSD molecules with AMPAR were uniformly distributed over 4–8 k_B_T. We considered the possibility that synaptic activity causes PSD molecules or receptors to be modified so that they can bind to each other at random times. As previously shown in Fig. 1, when all molecules exclusively act as obstacles, AMPAR diffusion is initially anomalous and receptors are trapped in the PSD (P = 0 in Fig. 5A). As the fraction of PSD molecules that can bind to AMPAR increases, there is a resulting increase in the net mobility of AMPAR; this is evident as an increase in the MSD of AMPAR (Fig. 5A). A logarithmic transform of the data shows that for even a small fraction of activation of PSD molecules (P>0.2) results in diffusion of AMPARs that is almost normal, albeit slow (Fig. 5B, with a small period of anomalous diffusion in the first 10 ms). This high sensitivity to the degree of binding is clearer in the plot of the anomalous exponent as a function of the fraction of activated PSD molecules (Fig. 5C). Thus, binding to PSD molecules increases the mobility of AMPARs otherwise trapped by molecular crowding and the diffusion process resulting from this binding to PSD molecules is practically normal.

We compared the results shown in Figs. 5A–C with simulations in which all the PSD molecules could bind to AMPARs (P = 1) with variable binding energies. Figure 5D shows a plot of MSD versus time for simulations with binding energies (E) ranging from 1 to 10 k_B_T. The logarithmic analysis shows that although anomalous diffusion is present over a short period of time, AMPAR diffusion returns to a normal process characterized by tortuosity (D_free_>D_app_ = constant) (Fig. 5E). As binding strength increases there is a delay in the transition from anomalous to normal diffusion (not shown) [49]. This is due to the effect of binding that traps an AMPAR in a single position in space for a period of time. However, this effect is strong only with large binding energies (E>10 k_B_T). Note that since this behavior resulted when all molecules covering 45% of the PSD bind strongly to AMPAR we consider this result to be an extreme case. As has been previously shown, the strength of the binding increases the cross-over time from anomalous to tortuous diffusion [49]. A linear fit to the late part of the diffusion process, indicated by the thick lines in Fig. 4E, results in a value of α close to 1 for all binding energies (Fig. 5F). It is important to note that although diffusion is normal it is very slow compared to AMPAR diffusion over a membrane without obstacles. Thus, a PSD in which all the molecules bind to AMPARs cannot reproduce the non-linear relationship between MSD and t observed in experimental results and, for a physiological range of binding energies, results in normal diffusion in less than 1 sec.

We next studied the general principle of how transient alterations in binding of PSD molecules can affect the concentration of AMPAR in the context of molecular crowding. Such reactions can be very complex, such as in long term potentiation (LTP) or depression (LTD) [50], [51], [52], [53]. We assumed that transient post-synaptic activation results in a post-translational modification - of either the C-terminal domains of the receptors [54] or the scaffold proteins in the PSD [11], [50], and that this transformation enhances the binding of PSD molecules to AMPAR. In order to determine a baseline influence of crowding on AMPAR retention after synaptic stimulation we assumed that a random subset of PSD molecules was allowed to bind to AMPAR with the same value of binding energies. After the period of synaptic activity ended, the active PSD molecules lost their ability to bind and returned to being obstacles.

**Figure 6:**
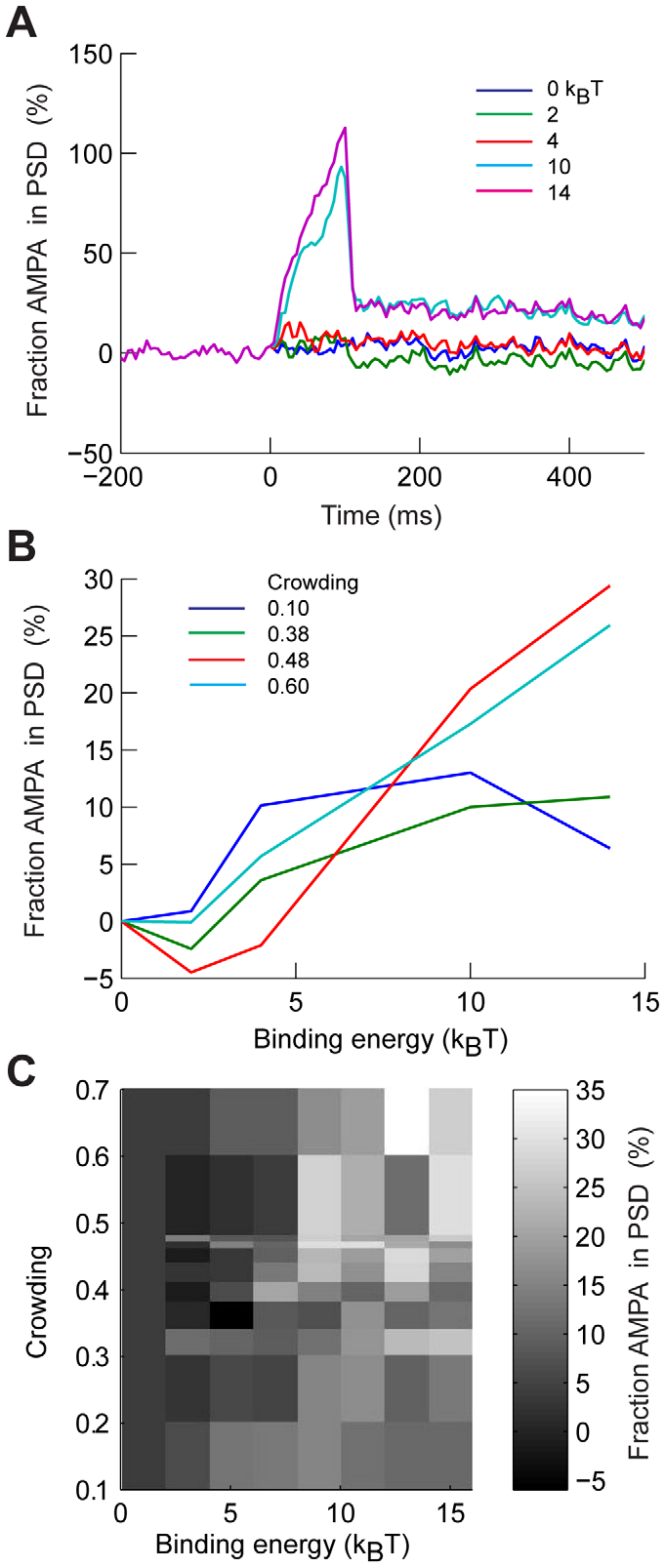
Effects of activity dependent binding and crowding on AMPAR retention in the PSD. We modeled 500 AMPARs diffusing in 2×2 µm membrane with a 0.5×0.5 µm PSD. For each level of crowding we homogeneously varied the binding energy of 10% of all PSD molecules to AMPAR over a 100 ms period, after which stimulation was stopped and AMPARs continued diffusing. **A**: Relative change of AMPARs found at the end of the simulation with respect to initial number inside the PSD as a function of time for different values of binding energies with C = 0.44. **B**: Relative change of AMPARs found at the end of the simulations with respect to initial number inside the PSD as a function of binding energies for three different crowding conditions. **C**: Summary gray scale plot of the change in AMPARs inside the PSD after stimulation for all values of binding and crowding explored. The simulations consisted in 500 ms of initial diffusion, 100 ms of stimulation, and 700 ms of diffusion after stimulation.

We simulated a large PSD that occupied a square area of 0.5×0.5 µm over a 2×2 µm patch of membrane. We explored the influence of a wide range of molecular crowding, from 0.00–0.60, and of binding energies, from 0–14 k_B_T. For each combination of molecular crowding and binding energies we ran 1000 different simulations in which an AMPAR was randomly placed in the membrane. After each set of 1000 simulations we calculated the percentage change of AMPARs found inside the PSD before and after the stimulation. Each simulation consisted in 500 ms before the stimulus, 100 ms of stimulation, and 700 ms after the stimulus, we calculated the average concentration in the first and last 400 ms of the simulation. The stimulus randomly activated only 10% of all the PSD molecules. Accumulation of AMPARs in the PSD depended on the amount of molecular crowding and strength of the stimulus. Figure 6A shows several traces of the relative concentration of AMPARs in the PSD as a function of time when C = 0.44 for different binding strengths. At a binding energy of 2 k_B_T more AMPARs left the PSD than were absorbed. As the binding strength increased AMPAR started to accumulate in the PSD. Figure 6B shows examples of the dependence of the fraction of AMPAR accumulation in the PSD as a function of binding for three different crowding values. The summary plot presented in Fig. 6C shows that for low values of binding strength and crowding, there is no accumulation of AMPARs. At high values of molecular crowding and binding there is always an increase of AMPAR in the PSD. However, there a transition region in which combinations of crowding and binding results in a slight depletion of AMPARs in the PSD. In this transition region the trapping of AMPARs is weakened by the increased net mobility of AMPARs due to binding. However, the low binding energies are not enough to recruit more AMPARs into the PSD. Increasing the fraction of PSD molecules binding to AMPAR would transform the PSD into a high-capacity buffer with slow kinetics, ultimately trapping more AMPARs. Thus, binding energies and molecular crowding result in different patterns of AMPAR accumulation in the synapse; after activity is terminated, molecular crowding retains the receptors within the PSD structure for long periods of time.

Taken together, our results suggest that, under conditions of molecular crowding and anomalous diffusion, steric interactions can have a significant effect in AMPAR retention inside the PSD. Steric interactions in combination with molecular binding can provide synapses the ability to retain AMPARs for prolonged periods of time and the flexibility to allow stimulus evoked AMPAR trafficking with the surrounding membrane.

## Discussion

Experimental evidence suggests that AMPAR diffuse non-linearly in the PSD and our simulations based on fundamental biophysical principles replicates these observations. Our model showed that the diffusion and retention of AMPAR in the PSD could be strongly affected by molecular crowding, which arises as a consequence of the high density of macro-molecules found in the PSD. Our simulations suggest that macro-molecular crowding within the PSD can result in anomalous diffusion of AMPAR, a process fundamentally different from diffusion in viscous or tortuous media. Thus, diffusible AMPARs could be retained inside this structure for long periods of time without the need of strong and prolonged binding. Counter-intuitively, but supported by experimental results, the binding of AMPAR to PSD molecules could serve to increase the net AMPAR mobility within synapses. The increased mobility is a consequence of the fundamental biophysical properties of protein diffusion and reaction on membranes. The functional consequence of the combination of trapping of AMPAR by molecular crowding and mobility by binding to PSD molecules results in the capability to regulate AMPAR transport in and out of the PSD.

### The biophysical properties of AMPAR diffusion on the plasma membrane

There are three fundamental physical assumptions common to all membrane protein diffusion studies that apply to the diffusion of AMPAR in and out of the PSD. First, when diffusing in an obstacle-free membrane AMPARs undergo normal diffusion (eq. 1). Second, AMPARs bind non-covalently and reversibly with PSD molecules [55]. Third, an elastic collision occurs if an AMPAR encounters, but does not bind to, a PSD molecule.

There is extensive experimental data showing that AMPAR movement in the extra-synaptic membrane can be described as an elastic random walk with elastic collisions [9]. This is plausible in neuronal membranes since water molecules are the main carriers of energy and there are more collisions between water molecules and proteins than between proteins. In any case, energy loss between collisions would only further the observed effects by decreasing the diffusion of AMPAR molecules.

The presence of membrane anchored proteins acting as obstacles for membrane protein diffusion has been documented in other cell types [56]. Since the PSD consists of a dense assembly of transmembrane and submembranous scaffold proteins, these proteins could sterically interact with AMPARs or other molecules through their C- or transmembrane-domains. For example, a variant of NCAM has been shown not to accumulate in the PSD [13]. However, the diffusion of NCAM is determined by the different splice variants which have different cytosolic domains. NCAM has been shown to undergo normal or anomalous diffusion depending on which one of the three splice variants is being studied [57]. Although, the NCAM data supports the hypothesis that steric interactions with the C-terminus are more important than collisions with transmembrane proteins in the PSD there are other forms of compartmentalization than arise from interactions with the dense extra-cellular matrix [45].

Since steric interactions are due to the physical presence of molecules and is not dependent on their identity, the diffusion of AMPAR should be affected by the total concentration of all macro-molecules. Even though a single molecular species might be regularly distributed over the PSD, the collection of all molecules could generate a dense mesh that effectively constitutes a random distribution of macro-molecules [17], [18]. Our simulations show that if steric interactions exclude AMPAR from diffusing in even 30% of the synaptic area, then net receptor diffusion will be severely hampered. The total mass of a 360 nm diameter and 30 nm thickness PSD has been calculated to be 1.1±0.36 GigaDaltons (or 1.83×10^−15^ gr), with a volume of 3.06×10^6^ nm^3^ [58]. The average protein density for macro-proteins is assumed to be constant at around 1.4×10^−21^ gr/nm^3^ [59], thus a solid PSD would contain 7.18×10^−15^ gr. Therefore, the fraction of PSD occupied by macro-molecules is (1.83×10^−15^/4.27×10^−15^) 43% by mass. The volume occupied in a PSD with the same dimension can be calculated by using the assumption that in a PSD there are 10,000 molecules of 100 kD [3], which results in an occupied volume of 50% (radius of a molecule is [(0.75/π) M.W./(1.4e−21 gr/nm^3^ * A)]^1/3^, with A being Avogadro's number, and M.W. the molecular weight). Although, not all cases of molecular crowding necessarily result in anomalous diffusion [60], the experimental measurements show that the PSD has the levels of molecular crowding necessary to observe the anomalous diffusion effects proposed by our model.

An alternative structural mechanism to confine receptor diffusion is the picket-and-fence model [61]. This model suggests that anchored molecules have specific arrangements that can trap molecules [10], [24], however, there is no anatomical evidence showing a picket-and-fence structure in the PSD. Furthermore, all experimental evidence shows that AMPARs execute a random walk on the extra-synaptic membrane or inside the PSD [10], [62], [63], [64]. Our work, as well as other computational studies [65], have shown that a picket-and-fence model does not produce the considerable anomalous diffusion observed for AMPAR.

### The properties of AMPAR-scaffold interactions

Dissociation constants for binding of AMPAR, or transmembrane AMPA receptor regulatory proteins (TARPs), such as stargazin, to the C-terminal domain binding proteins (CTDBP), such as GRIP, PICK and MAGUK proteins such as PSD-95, are in the range of 1–10 µM [48], [66], [67]. These values are in the upper part of our range of 2–13 k_B_T (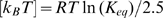, where *R* is the gas constant, *K_eq_* the equilibrium constant and 2.5 converts from kJ/mol to k_B_T units). Although no data is available to determine the percentage of PSD molecules that might bind to AMPAR or their strength, our simulations predict that changes in only a few of them are necessary to flip the ‘molecular switch’ from AMPAR retention to AMPA diffusion. These interaction energies may be directly measured using optical trapping techniques, as for the case of diffusing cadherin and transferrin receptors [68]. Also, in our simulations we assumed a uniform distribution of binding energies which might not be the case in a crowded PSD [17]. Deviations in the distribution of binding energies for PSD proteins would result in either higher or lower numbers of binding PSD needed to allow AMPAR mobility.

Although AMPAR-TARP interactions can be disrupted by glutamate [69], our results show that the observed increased mobility after bath application of glutamate of AMPAR inside the PSD (see Fig. 3 in [24]) could be a consequence of binding to static PSD molecules. The biophysical property of this assumption is the isotropic release of AMPAR after unbinding from a PSD molecule. As our other hypotheses, this assumption is not exclusive to our model. The well-stirred assumption, used to build and analyze all mass-action models of synaptic plasticity, implicitly assumes no directionality of binding and unbinding. Although this hypothesis is rarely addressed elsewhere, there are two physical properties of molecules that support such an assumption. The first one is the well-documented property of rotational diffusion (D_rot_) [70], [71], [72], [73]. The characteristic times of D_rot_ range from 10–100 µs. Rotational diffusion in combination with conformational changes can significantly modify the diffusional environment of diffusing molecules in the PSD. Molecular aggregation can modify this rotation to the point of stopping it [74], [75]. A different assumption that results in an average effective isotropic release after binding is the random orientation of the binding site of a population of anchored molecules. However, under conditions of molecular crowding the probability of interacting with the binding site can be severely reduced and the mobility of AMPAR would not vary in the presence or absence of binding. We assumed that isotropy of release was due to rotational diffusion. The major consequence of this assumption is that after unbinding, AMPARs can continue traveling beyond the point that was previously forbidden due to the volume exclusion imposed by the PSD protein. The combined effects of molecular crowding and binding can regulate the density of AMPAR in the PSD in such a way that molecular crowding retains the AMPAR diffusion in the absence of synaptic activity while binding allows their movement. Unfortunately, measurement of rotational diffusion has not been performed on AMPARs or any PSD molecules. Such measurements would determine whether the PSD behaves as a solid structure or if it is a crowded, yet mobile, system.

### Experimental validation

Experimental evidence suggests that the PSD is a tight mesh of proteins that can be considered stationary [76], [77], [78]. In general, our model predicts that by changing the concentration of non-interacting PSD molecules AMPAR residence time in the PSD could be modified. Our results can be tested by studying the dynamics of receptor mobility in synapses where both AMPARs and PSD scaffold molecules (such as PSD-95) are fluorescently labeled, with super-ecliptic phluorin (SEP-GFP) or photo-activable GFP and mCherry respectively. Monitoring the fluorescence recovery after photobleaching or the loss of fluorescence after photo-activation will yield a measure of the diffusivity of AMPARs, correlated with the amount of scaffold proteins (as measured by fluorescence intensity of mCherry-PSD-95). The amount of PSD scaffolds can be modified by manipulation of various protein-protein interaction domains of PSD-95 that mediate its retention in the synapse [79]. Finally, the role of specific interactions between receptors and scaffolds can be studied by observing the dynamics of transferrin receptors labeled by SEP-GFP (which marks membrane receptors). Since transferrin receptors are not known to interact with PSD-95, their mobility will allow the assessment of the contribution of crowding to receptor retention without being confounded by specific binding interactions. The role of binding interactions can be directly assessed by high resolution analysis of single particle tracks of receptors where direct interactions between receptors (or associated proteins) and scaffolds have been abrogated [11]. While this latter work did report increases in diffusivity of receptors when receptor-scaffold interactions were abolished, it was not clear whether this was due to lack of entry of receptors into synapses (purely extra-synaptic diffusion) or due to the fact that receptors entered the synapse but were not retained.

Measuring rotational diffusion of AMPARs or non-interacting molecules, such as NCAM, in the synaptic and extra-synaptic space would provide valuable information. We hypothesize that when AMPAR binds to scaffolding molecules its D_rot_ will decrease, thus measurements of D_rot_ in time would show changes from high to low values. However, if the receptors are in a crowded environment without binding then we expect to have a similar distribution of the values of D_rot_ inside and outside the synapse.

### The biological consequences of steric interactions in the PSD

#### Molecular crowding as a low-energy mechanism to retain AMPAR in the PSD

The notion of synaptic stability requires the retention of AMPARs for long periods of time [1], [12], [80]. Traditionally, the retention of AMPARs in the PSD is believed to be most strongly affected by biochemical interactions between receptors and PSD scaffolds [12]. However, all non-covalent binding is accompanied by a probability of unbinding [47], [81]. The highest binding energy among macro-proteins in the PSD is around 13 k_B_T, assuming a characteristic attempt rate of 1 µs results in an expected time to unbind of 0.44 ms (1×10^−6^/e^−13^). In the absence of steric interactions these AMPARs could escape the PSD if they are not bound to another anchored molecule. Steric interactions and molecular crowding could improve the retention of AMPARs in the PSD by considerably reducing the diffusion coefficient and thus increasing the probability of binding to another PSD molecule.

As noted above, the binding time of AMPAR to any PSD scaffold protein is much smaller than the characteristic time expression of LTP or LTD (minutes to hours). Our models suggest that molecular crowding, in conjunction with other cellular mechanism of synaptic homeostasis [82], could retain molecules for periods of seconds to hours. Under this model, the functional properties of the PSD are not only due to their specific biochemical composition but its density and stability, two characteristics that are preserved throughout evolution [83]. By maintaining a high PSD density a synapse could retain AMPARs for periods of time much longer than those provided by binding to scaffolding molecules. This *zero-energy model* of AMPAR retention in the PSD requires no extra energy from the synapse to retain a high density of AMPARs over long periods of time.

We hypothesize that plasticity mechanisms work on top of the basic biophysical mechanisms of receptor diffusion in a crowded PSD. For instance, phosphorylation of receptors, may allow a diffusing receptor to bind to PSD molecules via weak PDZ-domain interactions and enter the synapse. Even if the receptor is subsequently dephosphorylated, it can remain trapped in the synapse by crowding. Secondly, while experimental evidence suggests that the PSD is a tight mesh of proteins that can be considered stationary [76], [77], [78], synaptic plasticity has been shown to change the size of the PSD [77], [84], [85], [86], [87], [88]. If there is an enlargement of PSD area that preserves the number of molecules but decreases crowding, then our models predict that AMPAR could more easily enter and leave the PSD. If, however, PSD enlargement results from the aggregation of more molecules - with or without increasing their concentration - then this would result in longer residence times for AMPARs in the PSD. Our models predict that relatively small changes in molecular crowding determine whether AMPAR escape or remained trapped inside the PSD. In general, our model predicts that by changing the concentration of non-interacting PSD molecules AMPAR residence time in the PSD could be modified.

In order to maintain a constant density of AMPARs in the PSD there should be a constant number of PSD molecules ready to bind AMPARs [12] or changes in homeostatic cycling of AMPAR through endocytosis, exocytosis, and recycling [82]. For the timescales considered in this work, we assumed that the total number of extra-synaptic receptors is at a steady-state level set by the balance between exocytosis and endocytosis. Constitutive recycling occurs over a time scale of several minutes [89] and is consequently is a process that does not affect the overall level of receptors on a second time scale. Similarly, activity dependent insertion of receptors [5], [90], [91], [92] while rapid, takes several seconds.

#### Reduction in signal-to-noise ratio due to anomalous diffusion

Assuming a well-mixed PSD reduces the computational capacity of the synapse. In a well-mixed PSD all AMPARs have the same probability of binding with any other activate PSD molecule and their position within the PSD is not important. Such a process reduces the capacity of encoding synaptic activity to the total current generated by the activation of the glutamate receptors. If a PSD contains a small number of AMPARs then the encoding of the synaptic signal is inherently noisy and it is assumed to be averaged over multiple trials or multiple synapses [93]. However, in a crowded PSD where AMPAR are non-homogenously distributed and cannot achieve a steady state the reaction and encoding of synaptic activity could be different and potentially more reliable [94], [95].

Anomalous diffusion due to molecular crowding results in an increase in the correlation of the particle position along time, the appearance of long correlations can be measured using fluorescence correlation spectroscopy [30], [33], [96], [97]. On the contrary, in a well mixed system the temporal correlation among particles decays to zero as a function of D_free_ [29]. The increase in correlation results in an AMPAR visiting some sites more than others in the PSD compared to a homogenous random walk [18]. Thus, one AMPAR could sample one PSD molecule more times, thus increasing the reaction rate with that molecule [98], [99], [100], [101], [102]. Molecular crowding effectively creates nano-domains in the PSD that could be used by AMPAR to better detect glutamate signals [94], [103]. Retention in nano-domains within the PSD could result in higher encoding of the glutamate signal and enhanced transduction to specific scaffolding messaging molecules and could be the biophysical foundation of the high information encoding rates observed in biochemical systems [104], [105].

### Summary

Overall, our modeling efforts suggest that molecular collisions can keep AMPAR inside the PSD without requiring extra energetic processes [42] other than keeping the PSD crowded. This zero-energy model means that the PSD can maintain a difference in the concentration of AMPARs with respect to the surrounding membrane over long periods of time. Since NMDA receptors also undergo lateral diffusion then we expect the same processes to apply to their concentration in the PSD [106].

## Materials and Methods

### Monte Carlo simulation of diffusion

The average spatial displacement of a particle diffusing in a two dimensional membrane is described by
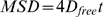
(1)Where t is time, D_free_ is the diffusion coefficient, and MSD is the average mean square displacement of the particle. The instantaneous MSD can be calculated over an ensemble of multiple independently diffusing particles,
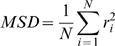
(2)Where r_i_ is the relative position of particle i to its initial position at time t, and N is the number of particles.

The membrane model consisted of a square mesh with toroidal boundary conditions. Particles crossing over the edge of the diffusing space would appear on the opposite edge in the next time step. The size of the rectangular mesh is specified in each simulation, but it was at least equivalent to 1 µm in side. The simulations were fully characterized by the time step Δt and the diffusion coefficient of AMPAR in the extra-synaptic membrane which we assume is the closest to an unobstructed system (D_free_ = 0.200×10^−3^ µm^2^/ms which results in a median D_free_ = 0.138×10^−3^ µm^2^/ms; [10]). At every time step the particle could move in any of the four directions defined in the rectangular mesh. In order to achieve this, a random number was drawn from a homogenous distribution to determine the axis of movement, and a second homogeneous random number was drawn to determine the direction of movement along the chosen axis.

The physical size of the mesh was determined using the expected displacement of a molecule given a Δt = 1×10^−3^ ms
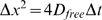
(3)which resulted in a value of Δx = 8.9×10^−4^ µm. This mesh size did not affect the calculation of the diffusion coefficient or the overall results of this study since performing the same simulations with 1000×D_free_ resulted in the same values of anomalous diffusion with only a rescaling of the simulation time [18].

### Modeling the PSD

The PSD is a disk-like structure composed of several thousand molecules that is 200–800 nm in diameter and 30–50 nm thick [3], [107]. Most of the PSD molecules are a few nanometers below the post-synaptic membrane, with some transmembrane protein complexes. The spatial arrangement of the PSD facing the membrane is smooth compared to the cytoplasmic side [108]. Since the life time of PSD molecules is longer than the synaptic plasticity effects studied here [109] and have low mobility [65], we consider them as essentially static [76], [77], [84], [85].

Based on the aforementioned properties, we modeled the PSD molecules as particles that did not diffuse and therefore occupied a single fixed position in the square mesh. In our algorithm a PSD was represented as an occupied point in the lattice. Diffusing molecules could collide with a static PSD molecule. A collision resulted in the AMPAR returning to its original position [65].

### Modeling molecular binding

Binding between large biological molecules occurs mainly through non-covalent bonds. Most protein-protein interactions are mediated by hydrogen bonds and van der Waals interactions [47]. The range of binding energies of hydrogen bonds is from 1–13 k_B_T and less than 1 k_B_T for van der Waals [47], [83]. We modeled the binding of diffusing AMPARs to scaffold proteins in the PSD as a stochastic second-order reaction. An AMPAR that moved into the position occupied by a PSD molecule had an initial probability of bouncing off p_bounce_.
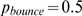
(4)If the AMPAR succeeded in binding to the PSD molecule, then the probability of remaining bound was given by an exponential potential
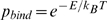
(5)Where k_B_ is the Boltzmann's constant and T is the temperature in Kelvin. Binding to scaffolding proteins could be a multi-order process; however, in this work we approximated binding with a single exponential energy barrier. As a reference, the 3 hydrogen bonds that make up a PDZ domain-ligand interaction have a total binding energy of about 10 k_B_T [81]. After binding, the AMPAR molecule remains fixed in that position until another homogenously distributed random number 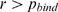. After unbinding we assumed that the molecule has an equal probability of moving in any direction. There are three possibilities that can determine the movement of AMPAR after unbinding: 1) AMPAR undocks and diffuses in the half-plane defined by the PSD molecule to which the AMPAR was bound; 2) conformational changes of the underlying PSD molecules remove the steric interaction, thus allowing AMPAR to move freely in any direction; 3) rotational diffusion of the AMPAR-PSD complex allows AMPAR to move in any direction. The rotational diffusion of cell membrane complexes is well known and has characteristic time constant ranging from 10–100 µs [70], [71], [72], [73]. In general, we assumed an isotropic direction of movement upon unbinding. Under the isotropic release paradigm, the newly freed AMPAR could move to any of the neighboring mesh points as long as they were un-occupied by another PSD molecule. Each simulation was independent; therefore, we did not model AMPAR-AMPAR interactions.

### Analysis

A typical simulation consisted of running 200–1000 AMPARs over the same membrane model with different initial conditions of the random number generator. We recorded the position of all the simulated particles every 1 ms. We tracked the spread of AMPARs from their point of origin at t = 0 over for up to 2000 ms [13].

### Implementation

The models were implemented using Matlab (Natick, MA) in combination with Star-P (Interactive Supercomputing, Waltam, MA). Star-P allowed us to utilize and run the original Matlab model in parallel at the Computational Biology Initiative high performance cluster at UTSA (http://www.cbi.utsa.edu).

## Supporting Information

Text S1 Supplementary Information
(0.27 MB PDF)
