# Supplementary material for: Quantifying the Effects of Elastic Collisions and Non-Covalent Binding on Glutamate Receptor Trafficking in the Post-Synaptic Density
Source: PLoS Comput Biol. 2010 May 13;6(5):e1000780. doi: 10.1371/journal.pcbi.1000780 (PMC2869312; doi:10.1371/journal.pcbi.1000780)
Supplement: Text S1 — Supplementary Information (0.27 MB PDF) [file pcbi.1000780.s001.pdf]

Supplementary Material

Quantifying the effects of elastic collisions and non-covalent binding on glutamate receptor trafficking in the post-synaptic density

Abbreviated title: AMPA diffusion in the PSD

Fidel Santamaria[1,2], Jossina Gonzalez[1], George J. Augustine[3], Sridhar Raghavachari[3]

[1]Biology Department and Neurosciences Institute[2], The University of Texas at San Antonio, One UTSA circle, San Antonio, TX, 78249

[3]Duke University Medical Center, P.O. Box 3209, Durham, NC, 27710

Corresponding author: Fidel Santamaria. One UTSA circle, University of Texas at San Antonio, San Antonio, TX 78249. fidel.santamaria@utsa.edu

EFFECTS OF PSD STRUCTURE ON NATURE OF AMPAR DIFFUSION

Molecules within the PSD might have a more regular structure than the random distribution of molecules assumed in the main text [1]. We quantified the effects of regularity in the arrangement of these molecules via multiple approaches. Our first approach was to start with a regular pattern in the membrane that was repeated. Figure 1A-B shows two examples where regularly distributed obstacles were used to model a lattice-based PSD. Figure 1A shows a case where a ring of PSD molecules was regularly repeated across the membrane. Each ring consisted of a single layer of PSD molecules in the perimeter with different densities, at 100% occupancy the entire perimeter of the ring is occupied by PSD molecules. This is meant to approximate the structure postulated by Holcman and Triller (2006) and could represent a ring of transynaptic adhesion molecules [2]. Figure 1B shows a different case where the PSD molecules were arranged as concentric rings. For the two spatial arrangements of PSD molecules shown in Figs. 1A-B we varied the density of randomly placed PSD molecules on the rings from 0-75%, with 100% resulting in a solid ring. The plots of MSD versus time and the logarithmic analyses (Fig. 1C-F) show that diffusion decreased but remained normal (α = 1). In both cases, the amount of molecular crowding was low (C < 0.4) due to the particular regular arrangements of particles. In either case, these simulations show that a lattice-like presence of obstacles can reduce the spread of AMPARs but would not result in anomalous diffusion.

The analyses for the data shown in Fig. 1 are for a particular arrangement of PSD molecules. In order to generalize these results we programmed an

algorithm that started with a homogenous and regular distribution of molecules across the entire simulated membrane. The program added disorder by randomly selecting 10 % of the molecules and moved them stochastically one lattice point. We increasingly added disorder by iterating recursively on the resulting distribution of PSD molecules. The PSD initially consisted of interleaving rows of obstacles and diffusion-enabling space, but as the number of iterations that added disorder increased, these free areas became randomly obstructed (Fig. 2A). The algorithm also checked for overlapping molecules such that the simulated membrane would contain the same amount of obstacles. We calculated the effect of PSD density and noise-adding iterations on the diffusion of AMPARs. This constancy ensured that, for any given obstacle density, the resulting data only reflected the impact of the distribution of proteins in the PSD on receptor diffusion. We ran our simulations using three values of molecular crowding (35, 40 and 45 %) that showed the largest change in anomalous diffusion (Fig. 1 in the main text).

Figure 2B shows the plots of anomalous exponent as a function of number of disorder-adding iterations for the three constant values of molecular crowding we previously selected. Each data point is the average of 800 simulations in which the original ordered PSD was disorganized with a different initialization of the random number generator. As expected, anomalous diffusion was not observed when there was no disorder in the system (leftmost points in each plot). This was expected because the lack of disorder resulted in a homogeneous pattern of obstacles that guaranteed at least one direction of diffusion. As the disorder

increased (a form of entropy), some pathways previously available for free diffusion were blocked while others were opened; however, the net effect was to generate anomalous diffusion, evident in Fig. 2B as a decrease in α. This noise-dependent increase in anomalous diffusion was most prominent and rapid in the PSD containing an obstacle concentration of 45 % (green in Fig. 2B). In summary, our analysis suggests that the effect of disorder on diffusion is largely influenced by the system's obstacle concentration and by the lack of regularity in the arrangement of obstacles. The data thus indicates that the structure of the PSD, both in terms of its obstacle density and degree of the randomness of its obstacle components, can significantly impact the pattern of diffusion within the PSD. In conclusion, our modeling results show that a randomly arranged PSD with molecular crowding larger than C = 0.4 is capable of retaining AMPARs inside the PSD without the need for binding interactions between receptors and PSD molecules. The retention of AMPARs is not due to trapping in fully closed areas; instead it arises by molecular collisions that cause anomalous diffusion.

COMPARISON OF MODELS AND EXPERIMENTS

The model originally developed by Kusumi et al. (1999) is widely used to determine the diameter of the explored area by the diffusing particle and to calculate its diffusion coefficient. We used a least-squared error algorithm to fit the curves of MSD vs time from Figure 1 in the main text with Kusumi's model (see main text for equation). The resulting confinement ratios (L) and diffusion coefficients (D) show a strong dependence on molecular crowding, Figures 3B-C respectively. As expected, Kusumi's model correctly recovers the diffusion

coefficient of the simulation (0.2 μm^2/ms) with no molecular crowding. As we increase the value of C, the fitted diffusion coefficient decreases as well as the confinement ratio.

We then fitted several experimentally reported plots of MSD vs time with the anomalous diffusion equation (eq. 6 in main text). For each plot we digitized the values of MSD and t and used a least-squared error algorithm to fit the data (Figure 4). Each plot reports the values of the diffusion coefficient and anomalous exponent for each curve and the corresponding molecular crowding value. Remarkably, for diffusion inside synapses most of the fits resulted in crowding values close to C = 0 .44, while for extrasynaptic diffusion C ~ 0.40. In all cases, the values of the fitted diffusion coefficient are within the experimentally reported range [3].

REFERENCES

1. Ritchie K, Iino R, Fujiwara T, Murase K, Kusumi A (2003) The fence and picket structure of the plasma membrane of live cells as revealed by single molecule techniques (Review). Mol Membr Biol 20: 13-18.

2. Gorski JA, Gomez LL, Scott JD, Dell'Acqua ML (2005) Association of an A-kinase-anchoring protein signaling scaffold with cadherin adhesion molecules in neurons and epithelial cells. Mol Biol Cell 16: 3574-3590.

3. Ehlers MD, Heine M, Groc L, Lee MC, Choquet D (2007) Diffusional trapping of GluR1 AMPA receptors by input-specific synaptic activity. Neuron 54: 447-460.

FIGURE LEGENDS

Figure 1

Picket-and-fence models have a linear effect on reducing AMPAR. **A**-**B**: Renderings of a section of the model showing two types of regular arrangements of PSD molecules. The black dots show the position of a molecule. **C-D**: MSD vs t curves show that as the density of PSD molecules increases the diffusion of AMPAR is decreased. **E-F**: The logarithmic transform of the data in **C**-**D** show that the diffusion is reduced but remains normal. Each curve is calculated from 400 simulations.

Figure 2

Increasing random distribution of PSD molecules result in anomalous diffusion of AMPARs. **A**: The simulations consisted in calculating the diffusion of AMPAR over a PSD with toroidal boundary conditions. Initially, the PSD obstacles were homogeneously distributed by interleaving empty space with a row of obstacles of a given density. One iteration consisted in randomly selecting 10% of the PSD obstacles and randomly shifting them one lattice point. Every resulting new distribution of the PSD had the same number of crowding molecules (C). **B**: The plot shows the calculated value of the anomalous exponent (α) of AMPARs diffusing inside a PSD for different amounts of noise added to the position of the PSD obstacles. Each data point was calculated form 800 simulations. The error

bars are the S.E.M. for calculating the value of α over 6 different initialization of the random number that determined the structure of the PSD.

Figure 3

AMPAR diffusion is reduced by collisions with anchored PSD molecules. **A**: Plots of MSD vs time reproduced from Figure 1 in the main text (blue). Each plot was fitted with Kusumi's model (red). **B**: Value of confinement length (L) obtained from the fits in A as a function of molecular crowding. **C**: Value of diffusion coefficient obtained from the fits in A as a function of molecular crowding.

Figure 4

Anomalous diffusion fits to experimental data. Each plot was obtained from the indicated references. For each data set we fitted the anomalous diffusion equation (eq. 6 in the main text).

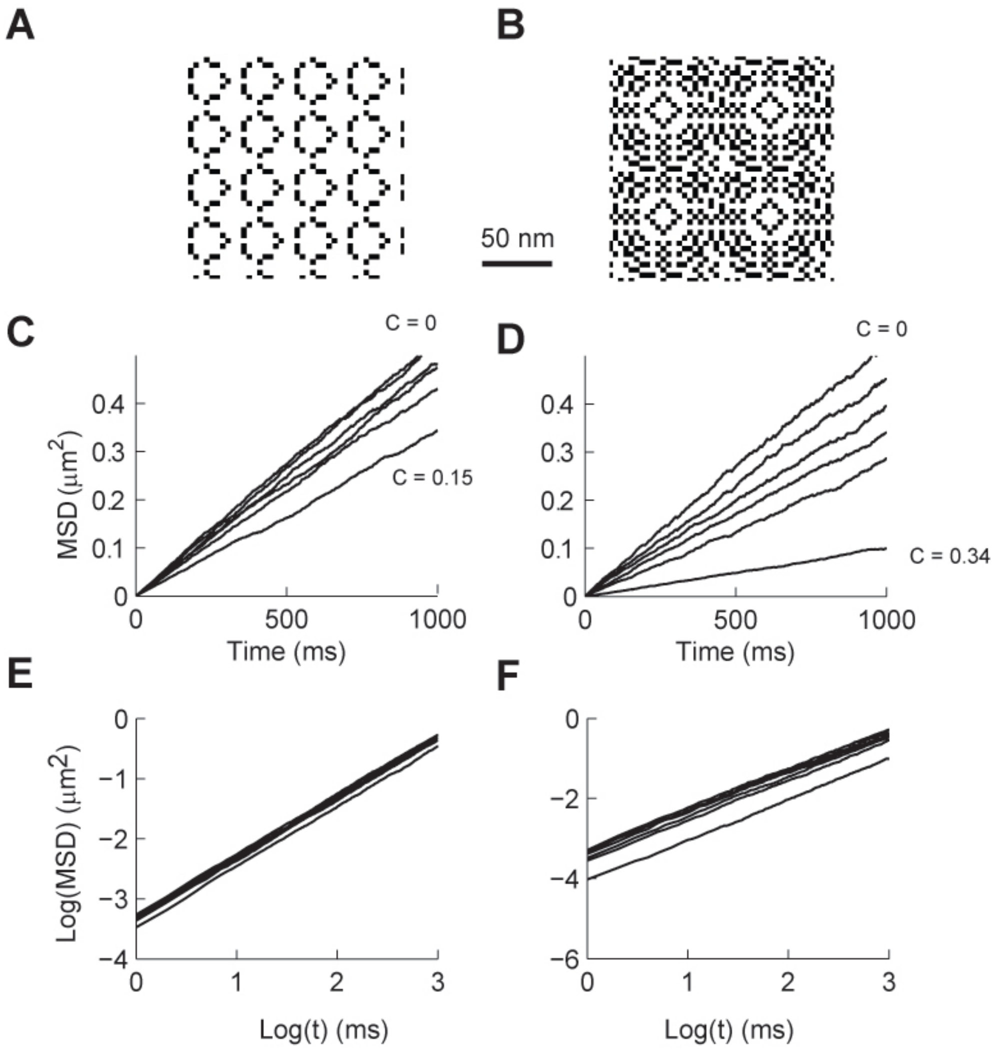
A
B
50 nm
C
C = 0
C = 0.15
MSD (μm^2)
Time (ms)
D
C = 0
C = 0.34
Time (ms)
E
Log(MSD) (μm^2)
Log(t) (ms)
F
Log(t) (ms)

Figure 1

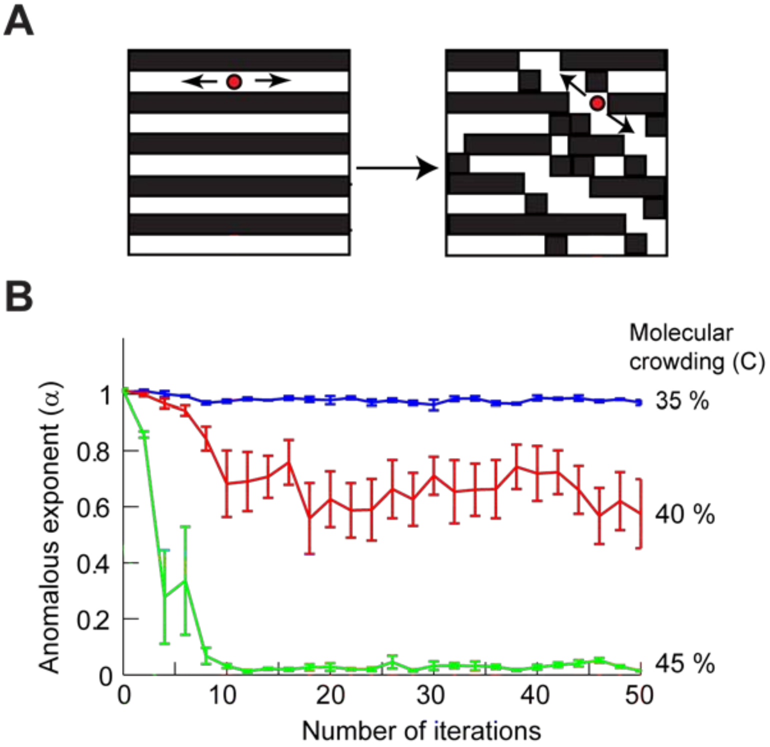
A
B
Molecular
crowding (C)
35 %
40 %
45 %
Anomalous exponent (α)
1
0.8
0.6
0.4
0.2
0
0
10
20
30
40
50
Number of iterations

Figure 2

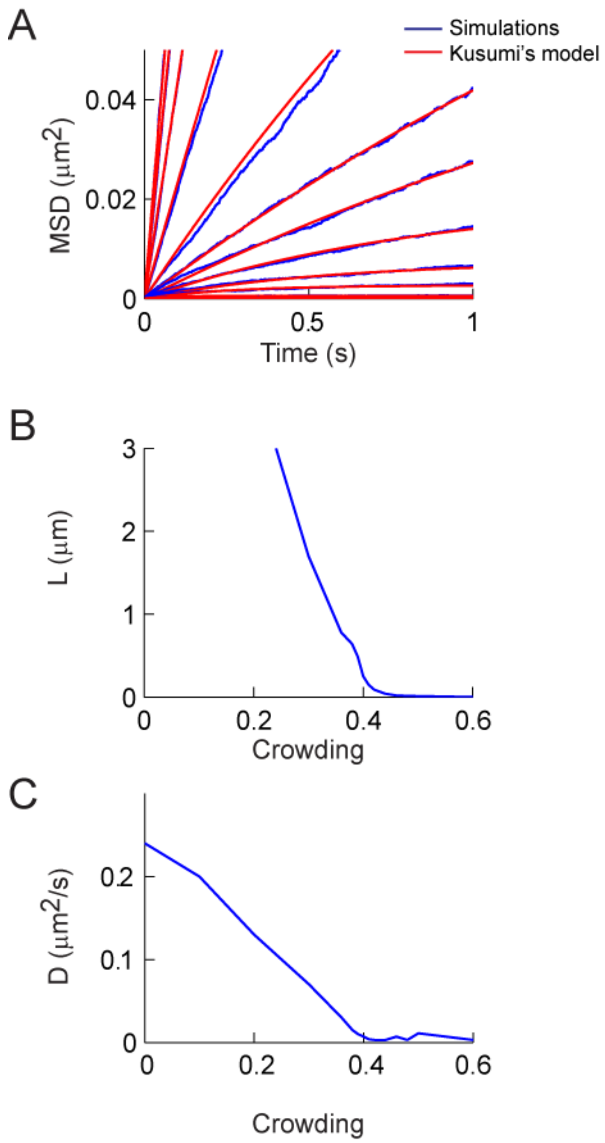
A
Simulations
Kusumi's model
MSD (μm2)
0.04
0.02
0
0
0.5
1
Time (s)
B
L (μm)
3
2
1
0
0
0.2
0.4
0.6
Crowding
C
D (μm2/s)
0.2
0.1
0
0
0.2
0.4
0.6
Crowding

Figure 3

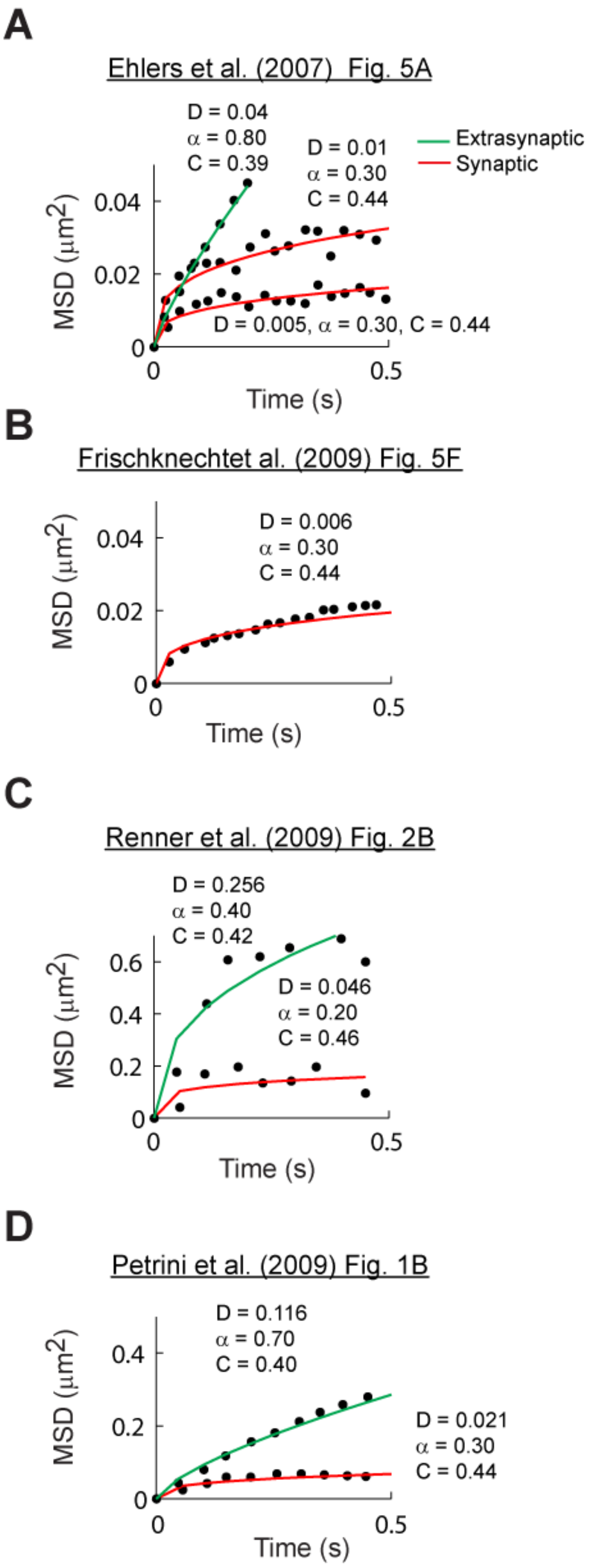
A
Ehlers et al. (2007) Fig. 5A
D = 0.04
α = 0.80
C = 0.39
D = 0.01
α = 0.30
C = 0.44
Extrasynaptic
Synaptic
MSD (μm2)
0.04
0.02
0
D = 0.005, α = 0.30, C = 0.44
0
0.5
Time (s)
B
Frischknechtet al. (2009) Fig. 5F
D = 0.006
α = 0.30
C = 0.44
MSD (μm2)
0.04
0.02
0
0
0.5
Time (s)
C
Renner et al. (2009) Fig. 2B
D = 0.256
α = 0.40
C = 0.42
D = 0.046
α = 0.20
C = 0.46
MSD (μm2)
0.6
0.4
0.2
0
0
0.5
Time (s)
D
Petrini et al. (2009) Fig. 1B
D = 0.116
α = 0.70
C = 0.40
D = 0.021
α = 0.30
C = 0.44
MSD (μm2)
0.4
0.2
0
0
0.5
Time (s)

Figure 4
